# Enhanced Production and Profiling of Ganoderic Acids in *Ganoderma lucidum* Mycelia via Two-Stage Cultivation and GNPS-Guided Metabolomics

**DOI:** 10.3390/jof12070500

**Published:** 2026-07-08

**Authors:** Chieh-Hsi Tsao, Hsin-Ya Tsai, Kai-Wen Cheng, Guan-Yuan Chen, Hao-Ting Chen, Cheng-Chih Hsu, Nan-Wei Su

**Affiliations:** 1 Department of Agricultural Chemistry, National Taiwan University, Taipei 10617, Taiwan; r10623010@ntu.edu.tw (C.-H.T.); d08623003@ntu.edu.tw (H.-Y.T.); r12623024@ntu.edu.tw (H.-T.C.); 2 Leeuwenhoek Laboratories Co., Ltd., Taipei 10688, Taiwan; d07223127@ntu.edu.tw (K.-W.C.); ccrhsu@ntu.edu.tw (C.-C.H.); 3 Department and Graduate Institute of Forensic Medicine, College of Medicine, National Taiwan University, Taipei 10617, Taiwan; gychen@ntu.edu.tw; 4 Department of Chemistry, National Taiwan University, Taipei 10617, Taiwan; 5 Department of Biochemical Science and Technology, National Taiwan University, Taipei 10617, Taiwan

**Keywords:** ganoderic acids, *Ganoderma lucidum*, GNPS, molecular network, mycelia, triterpenoids

## Abstract

Ganoderic acids (GAs) are bioactive lanostane-type triterpenoids produced by *Ganoderma lucidum* that accumulate predominantly in fruiting bodies, whose long cultivation period limits their practical production. Using Global Natural Products Social Molecular Networking (GNPS), we identified *G. lucidum* TM701, which accumulated 99 GA derivatives in mycelia and exhibited higher triterpenoid levels and greater chemical diversity than the commercial strain BCRC 36203. Four abundant GAs (GA-Mb/Mc, GA-S/Mf, GA-T, and GA-R) were selected as marker compounds for monitoring GA production. A modified two-stage cultivation strategy, combining submerged inoculum preparation with nutrient optimization during static cultivation, increased GA production to 1396 mg L^−1^ GAs in potato dextrose broth supplemented with 2% glucose. Heat treatment revealed interconversion among GA-Mb/Mc, GA-Mh, and GA-P/Q, indicating a dehydration-driven stabilization of conjugated diene structures. *G. lucidum* TM701 mycelia not only provide a platform for triterpenoid production, but also serve as a promising model for elucidating GA biosynthesis and investigating their thermal transformation.

## 1. Introduction

Ganoderic acids (GAs), the most abundant lanostane-type triterpenoids in *Ganoderma* spp., exhibit diverse biological activities, including anticancer, immunomodulatory, cardioprotective, and anti-HIV effects [1,2,3,4]. Some GAs also show inhibitory potential against the SARS-CoV-2 main protease [5]. However, the limited yield of GAs constrains their clinical and industrial applications [6]. Unlike pentacyclic triterpenoids from plants, tetracyclic triterpenoids in fungi are synthesized via the mevalonate (MVA) pathway, in which key enzymes such as 3-hydroxy-3-methylglutaryl coenzyme A reductase (HMGR), squalene synthase (SQS), and lanosterol synthase (LS) are expressed at low levels in mycelia [1,7]. Consequently, GAs predominantly accumulate in fruiting bodies, whose cultivation is labor-intensive and time-consuming [2,8].

Submerged mycelial culture has emerged as a promising alternative for triterpenoid production [9,10]. Various strategies, including genetic overexpression [6,11], inducer addition [12], and two-stage cultivation [9,13,14,15] have improved GA yield; however, achieving levels comparable to those in fruiting bodies remains challenging. While the triterpenoid composition of fruiting bodies has been well characterized [16], the specific GA species preferentially synthesized under mycelial culture conditions, as well as the strain-dependent metabolic variations among different *Ganoderma* strains, remain poorly understood. Moreover, identifying strains with intrinsically high triterpenoid-producing capacity is hindered by the vast structural diversity of GAs and their numerous analogs, which complicate triterpenoid profiling. Recent advances in MS/MS-based molecular networking, particularly Global Natural Products Social Molecular Networking (GNPS), have facilitated the annotation and comparison of structurally related metabolites [17]. GNPS has been applied to characterize triterpenoid profiles across different *Ganoderma* species [18] and to screen bioactive compounds [19] in fruiting bodies and spores. However, comprehensive metabolomic analyses focusing on strain-level differences in *Ganoderma* mycelial triterpenoid profiles remain limited.

Here, we identified *G. lucidum* TM701 as a high GA-producing strain capable of accumulating GAs in mycelia at levels surpassing those found in fruiting bodies. We applied untargeted metabolomics integrated with GNPS to profile triterpenoids in mycelial cultures of *G. lucidum* TM701 and the commercial strain BCRC 36203, and to investigate cultivation strategies that enhance GA biosynthesis as well as potential heat-induced interconversion among GAs species.

## 2. Materials and Methods

### 2.1. Chemicals and Biomaterials

Ganoderic acid A (GA-A, ≥98%, HPLC grade) was purchased from Sigma-Aldrich (St. Louis, MO, USA). HPLC-grade acetonitrile (ACN) and methanol were obtained from Merck (Darmstadt, Germany). Potato dextrose broth (PDB) and agar were supplied by Difco (Detroit, MI, USA), and wheat bran powder was kindly provided by JOY & HOPE (Yunlin, Taiwan). *G. lucidum* BCRC 36203 was obtained from the Bioresource Collection and Research Center (Hsinchu, Taiwan), while *G. lucidum* TM701 was originally isolated from a natural environment and provided by Wishsun Natural Farm (Hsinchu, Taiwan). The appearance of the *G. lucidum* TM701 fruiting body is shown in Figure S1. Species identity was verified by BLASTn analysis (https://blast.ncbi.nlm.nih.gov/Blast.cgi?PROGRAM=blastn&PAGE_TYPE=BlastSearch&LINK_LOC=blasthome accessed on 2 July 2026) of the internal transcribed spacer (ITS) region using primers ITS4 and ITS5 (Table S1). All strains were maintained on potato dextrose agar plates and incubated for 7 days at 30 °C until approximately 80% surface coverage was achieved. For seed culture preparation, 10 agar plugs (5 mm diameter) were transferred into 500 mL Erlenmeyer flasks containing 150 mL PDB and incubated at 30 °C, 100 rpm, in darkness for 7 days.

### 2.2. Two-Stage Cultivation of G. lucidum Strains for GA Accumulation

A wheat bran infusion was used as the first-stage medium. Wheat bran powder (100 g) was mixed with 1 L of distilled water, sterilized at 121 °C for 20 min, filtered through a 100-mesh sieve, and diluted to a total solid content of 2.4%. For the first-stage cultivation, 0.3 L of seed broth was inoculated into a 5.0 L stirred-tank bioreactor (Winpact FS-V-D05, Major Science Co., Ltd., Taoyuan, Taiwan) containing 2.7 L of the wheat-bran infusion. The dissolved oxygen level was maintained at ≥15% by adjusting agitation and aeration, and cultivation proceeded at 30 °C for 5 days. For the second stage, the resulting culture was inoculated (10%, *v*/*v*) into PDB medium with or without glucose supplementation and incubated statically in 10 cm diameter glass jars sealed with cotton plugs to allow gas exchange (Figure S2). The cultures were maintained at 30 °C and 80–85% relative humidity in the dark for 35 days. All experiments were performed with five independent biological replicates (*n* = 5). Mycelia were harvested, rinsed with distilled water, dried at 40 °C overnight, and ground to pass through a 20-mesh sieve. The dried powders were stored at −20 °C until further analysis.

### 2.3. Untargeted Analysis of Triterpenoids in Mycelial Cultures

Dried mycelial powder (100 mg) was extracted twice with 5 mL methanol under stirring for 30 min. The combined supernatants were centrifuged (10,000× *g*, 5 min), pooled, and adjusted to a final volume of 10 mL with methanol. Extracts were filtered through 0.22 µm membranes and diluted to 1000 ppm prior to LC–MS analysis. Untargeted profiling was performed using a Vanquish UPLC-DAD system (Thermo Fisher Scientific, Waltham, MA, USA) coupled to an Orbitrap Elite hybrid ion trap-Orbitrap mass spectrometer. Separation was achieved on a YMC-Triart C8 column (YMC Co., Ltd., Kyoto, Japan; 250 × 4.6 mm, 5 µm) using mobile phases of (A) 0.1% (*v*/*v*) formic acid in water and (B) ACN. The gradient program was 30–65% B (0–20 min), 65–75% B (20–30 min), 100% B (40–45 min), 100–30% B (45–55 min), and 30% B (55–65 min). The flow rate was 1 mL min^−1^, column temperature 30 °C, and injection volume 20 µL. UV absorbance was monitored at 245 nm as described by Tang et al. [20]. Mass spectra were acquired in negative ion mode with fragment ion mass tolerance ≤ 5 ppm (instrument parameters listed in Table S2). All samples were analyzed within the same LC-MS analytical batch under identical chromatographic and mass spectrometric conditions to minimize inter-batch variation.

### 2.4. Quantification of GAs

For quantification, 200 µL of methanolic extract was mixed with 50 µL of methanol containing GA-A (100 ppm) as the internal standard. LC-UV analysis was performed on a Shimadzu LC-10AD system (Shimadzu Corporation, Kyoto, Japan) equipped with a UV6000 photodiode-array detector (Thermo Fisher Scientific, Waltham, MA, USA) under the same chromatographic conditions as described above. Since authentic standards for all GA species were unavailable, each GA was quantified relative to a GA-A calibration curve (r^2^ = 0.999, Figure S3), and results were expressed as GA-A equivalents. Total GA content was calculated as the sum of all individual GA-A equivalent concentrations.

### 2.5. Determination of Biomass and Residual Sugar

Moisture content of the mycelial powder was determined using a Shimadzu MOC-63U infrared moisture analyzer (Kyoto, Japan) to obtain dry biomass. Residual sugar in the culture broth was measured using the 3,5-dinitrosalicylic acid method, with glucose (Sigma-Aldrich, St. Louis, MO, USA) as the standard. Absorbance was recorded at 570 nm using a UV-Vis spectrophotometer (U-5100, Hitachi High-Tech Corporation, Hitachinaka, Ibaraki, Japan).

### 2.6. GNPS Data Processing and Statistical Analysis

Raw LC–MS data were converted to mzXML format using MSConvert (ProteoWizard, version 3) to centroid spectra and uploaded to the GNPS platform (http://gnps.ucsd.edu; accessed on 3 May 2026) via WinSCP for molecular networking. Parent and fragment ion tolerances were both set to 0.02 Da, with cosine scores > 0.7 and ≥6 shared fragment ions required for node connection. Spectral files from TM701 and BCRC 36203 were assigned to Group 1 (G1) and Group 2 (G2), respectively, with three independent biological replicate LC–MS/MS files per group. Resulting molecular networks were visualized using Cytoscape v3.8 and are available online at: https://gnps.ucsd.edu/ProteoSAFe/status.jsp?task=f3a43a0ea4ff45ed9a6f3caceb1c95df (accessed on 3 May 2026). Compound alignment and annotation were performed in Compound Discoverer 3.2 using the mzCloud and ChemSpider databases. Quantitative data are presented as mean ± standard deviation (SD) from at least three independent replicates, and statistical analyses were conducted using Microsoft Excel 2019 and GraphPad Prism v9.5.1 (GraphPad Software, San Diego, CA, USA).

## 3. Results and Discussion

### 3.1. Strain Comparison and Metabolic Profiling of G. lucidum Mycelial Cultures

**Figure 1:**
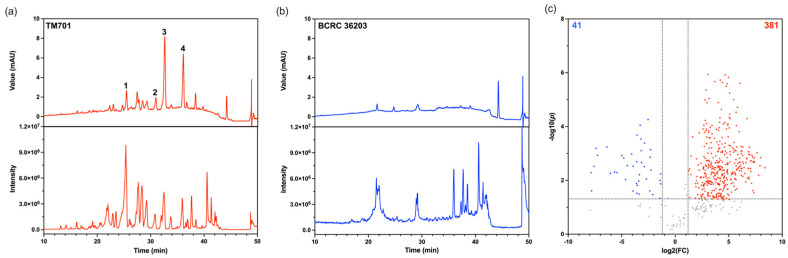
Comparative metabolite profiles and molecular networking of two *G. lucidum* strains. (**a**,**b**) LC-UV chromatograms (upper panel) and LC-negative ion ESI-MS total ion chromatograms (TIC, lower panel) of *G. lucidum* TM701 and BCRC 36203, respectively. (**c**) Volcano plot of differentially abundant triterpenoid species between *G. lucidum* strains TM701 (red) and BCRC 36203 (blue). The volcano plot is a combination of FC (TM701/BCRC 36203) and *t*-tests; the x-axis is log2 (FC), and the Y-axis is −log10 (*p*-value). Each dot represents one metabolite; red and blue dots indicate that they passed the threshold in both *t*-test (*p*-value < 0.05 and |log2FC| > 1.2). The volcano plot was constructed from three independent biological replicates for each strain.

*G. lucidum* is a well-known producer of GAs, a structurally diverse group of lanostane-type triterpenoids. Although strain-dependent variations in triterpenoid profiles have been reported in fruiting bodies [21], strain-specific differences in GA biosynthesis during the mycelial growth phase remain less explored. To evaluate the GA-producing potential of different strains, two *G. lucidum* strains, TM701 (isolated from a local farm) and BCRC 36203 (a commercial reference strain), were compared under identical cultivation conditions. Two-stage cultivation was employed, in which shake-flask cultures were grown for 7 days to generate inoculum, followed by transfer to fresh medium and 28 days of static incubation to stimulate GA accumulation in mycelia. LC–UV analysis showed that TM701 mycelial extracts contained more peaks with strong absorption at 245 nm, corresponding to the characteristic wavelength of GAs [20,22,23], as shown in Figure 1a,b. Quantitative analysis based on the total peak area at 245 nm revealed a substantially higher GA content in TM701 mycelia than in BCRC 36203. Using GA-A as an internal standard, the estimated GA content reached 87.9 mg g^−1^ dry weight (DW) in TM701, compared with 12.6 mg g^−1^ DW in BCRC 36203 (Table S3). These results indicate the superior GA-producing capacity of TM701.

**Figure 2:**
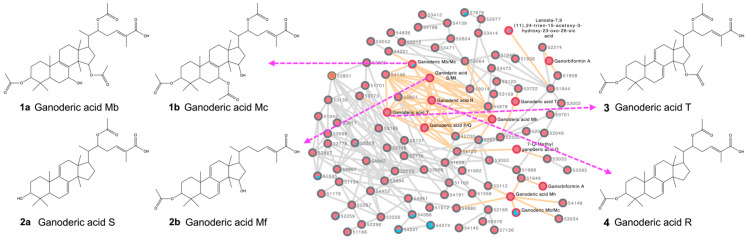
The major triterpenoid cluster from the MS/MS molecular network of *G. lucidum* strains TM701 and BCRC 36203 generated via GNPS. Node color indicates the strains in which the compound was found (coral: TM701; blue: BCRC 36203). Each node is displayed as a pie chart, with the color proportions reflecting the relative spectral counts contributed by each strain. Red-framed nodes represent identified compounds, with orange edges indicating their spectral similarity. Four nodes (GA-Mb/GA-Mc, GA-S/GA-Mf, GA-T, and GA-R) were selected as representative triterpenoid markers for subsequent experiments. Nodes with the highest structural similarity are positioned closer to the center, reflecting their central role in the cluster’s connectivity.

To further assess metabolic differences between the two strains, untargeted LC–ESI–MS/MS metabolomic data were analyzed using volcano plots (Figure 1c). A total of 381 metabolite features were significantly enriched in TM701, compared with 41 enriched in BCRC 36203 (*p* < 0.05, fold change > 2.3 or <0.43). These findings indicate pronounced metabolic divergence and suggest a higher potential for secondary metabolite biosynthesis in TM701. Structural relationships among triterpenoid metabolites were visualized using GNPS analysis, revealing 46 multi-node clusters (≥2 nodes) (Figure S4). The node IDs and corresponding precursor *m*/*z* values are provided in Table S4. One major cluster contained 99 nodes, with several highly connected nodes corresponding to known GAs, such as GA-Mb/Mc, GA-P/Q, GA-R, GA-S/Mf, and GA-T, suggesting this cluster represents triterpenoid-related metabolites (Figure 2). TM701 contributed a larger number and higher abundance of triterpenoid nodes than BCRC 36203, indicating greater triterpenoid diversity. Several unannotated nodes within the triterpenoid cluster likely represent other GA species with minor structural modifications. Collectively, these analyses suggest that TM701 produced more diverse triterpenoids than the commercial reference strain.

### 3.2. Identification of Triterpenoid Species in G. lucidum TM701 Mycelia

Compound annotation was performed by combining GNPS molecular networking with LC–MS/MS spectral interpretation, and an in-house reference database of *Ganoderma* triterpenoids compiled from published literature [16,20,21,24]. The reference database has been deposited in an Open Science Framework repository as an Excel file and is publicly available at: https://osf.io/rsvyx/overview?view_only=13d74a94312c455da85a79986e3ab6fd (accessed on 2 July 2026). Twenty-one triterpenoid species, including several isomers, were identified in TM701 mycelia (Figure S5). Based on the metabolite annotation confidence criteria proposed by Sumner et al., these compound annotations correspond to Level 2 (putatively annotated compounds), as they were assigned based on accurate mass measurements, MS/MS fragmentation patterns, and comparison with published literature [25]. Major constituents included GAs and ganolucidic acids bearing carboxylated side chains. Commonly reported triterpenoids, such as GA-P, GA-Q, GA-Mb, GA-Mc, GA-T, GA-S, GA-R, GA-Mh, 7-O-methyl-GA-O, and lanosta-7,9(11),24-trien-15α-acetoxy-3α-hydroxy-23-oxo-26-oic acid, were detected, consistent with previous reports [9,26,27]. In addition, ganolucidate F, GA-GS-2, GA-Jc, ganolucidic acid E, and GA-Jb, typically found in fruiting bodies, were present in TM701 mycelia, and ganolucidic acid D, previously reported in spores and fruiting bodies, was also detected [26].

Most of these compounds have documented cytotoxic or antiproliferative activities against cancer cells [28,29], underscoring the pharmacological relevance of TM701 metabolites. The detection of fruiting body–specific compounds in mycelia suggests that TM701 may express biosynthetic enzymes responsible for advanced oxidation and side-chain modification steps in GA synthesis. These findings support the potential of TM701 as a promising cell factory for triterpenoid biosynthesis. Among the identified metabolites, four major LC peaks, with retention times of 24.8, 30.97, 32.10, and 35.36 min, were assigned as GA-Mb/Mc, GA-S/Mf, GA-T, and GA-R, respectively, based on their MS/MS spectra (Figure S5). Due to their abundance and structural centrality within the GNPS network, these four GAs were selected as representative markers for subsequent optimization of culture conditions.

### 3.3. Nutritional Effects on GA Accumulation During Second-Stage Static Cultivation

To enhance mycelial biomass for GA production, a modified two-stage cultivation system was employed. In the first stage, submerged bioreactor fermentation was used to generate a high-density inoculum. Biomass and residual sugar profiles were recorded throughout the 5-day cultivation period, while pH and dissolved oxygen were continuously monitored (Figure S6). Biomass increased modestly during the first 48 h, then increased rapidly to 12.8 ± 0.3 g L^−1^ by day 5. Meanwhile, residual sugar concentrations remained stable initially but declined after 48 h, reflecting carbon utilization. PDB medium, a widely used medium for fungal culture, was selected as the base medium for subsequent static cultivation to evaluate the effects of nutrient composition on GA accumulation.

**Figure 3:**
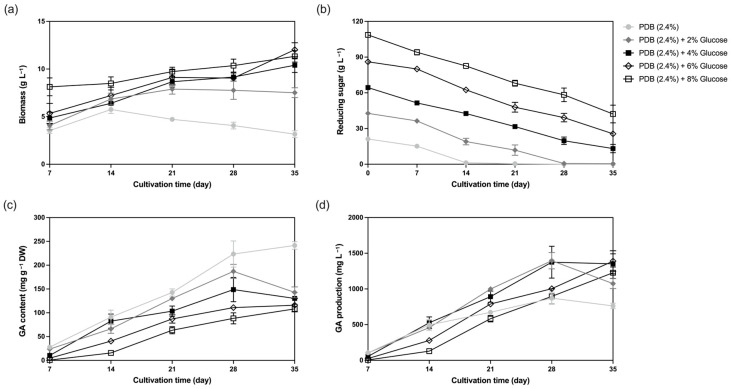
Time-course profiles of (**a**) biomass, (**b**) residual sugar, (**c**) GA content, and (**d**) GA production in PDB groups supplemented with 0–8% (*w*/*v*) glucose during the second-stage cultivation of *G. lucidum* TM701. Data are presented as mean ± SD (*n* = 5). The culture broth containing mycelia obtained from the first-stage cultivation as the inoculum was transferred 10% (*v*/*v*) to sterilized jars containing 45 mL of the second-stage cultivation medium, and then incubated at 30 °C with a relative humidity of 80–85% for 35 days. The GA content and production were calculated as the sum of all identified GAs, with each compound quantified and expressed as GA-A equivalents.

To investigate how nutrient availability influences GA biosynthesis, PDB media were supplemented with varying glucose concentrations (0–8%, *w*/*v*). As shown in Figure 3a, in the absence of glucose, the highest mycelial biomass was reached on day 14, whereas 2–6% glucose supplementation sustained growth for a longer period and resulted in higher biomass. At 8% glucose, growth plateaued, likely due to osmotic stress caused by the elevated sugar concentration (~11 atm) [30,31]. Regarding GA production, the highest level was achieved around day 28 in PDB containing 0–4% glucose, whereas higher glucose levels (6–8%) led to a more gradual and prolonged increase (Figure 3d). Maximum GA production was obtained with 2% glucose, reaching 1395.9 ± 114.8 mg L^−1^ at day 28, compared to 762.4 ± 37.7 mg L^−1^ in PDB without additional glucose supplementation. Although 2% glucose slightly reduced GA content per biomass (241.5 ± 8.0 mg g^−1^ DW), the total volumetric yield improved due to enhanced biomass growth. Further glucose addition (4–8%) did not improve GA production and may increase the risk of contamination due to residual sugars (Figure 3b).

**Figure 4:**
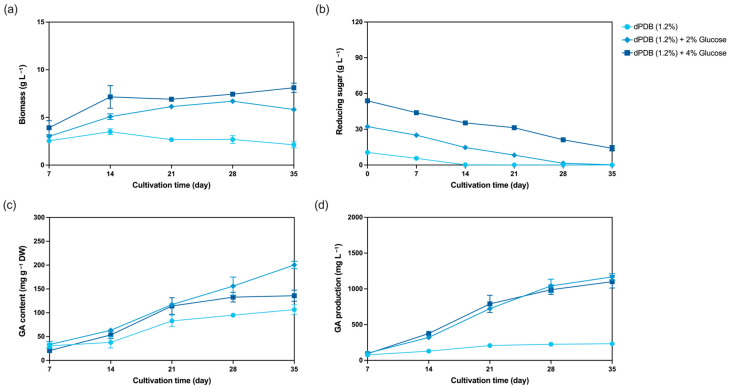
Time-course profiles of (**a**) biomass, (**b**) residual sugar, (**c**) GA content, and (**d**) GA production in dPDB (PDB diluted to 50%) groups supplemented with 0–4% (*w*/*v*) glucose during the second-stage cultivation of *G. lucidum* TM701. Data are represented as mean ± SD (*n* = 5). The culture broth containing mycelia obtained from the first-stage cultivation as the inoculum was transferred 10% (*v*/*v*) to sterilized jars containing 45 mL of the second-stage cultivation medium, and then was incubated at 30 °C with a relative humidity of 80–85% for 35 days. The GA content and production were calculated as the sum of all identified GAs, with each compound quantified and expressed as GA-A equivalents.

To evaluate whether nitrogen limitation could maintain GA production under reduced nutrient supply while also reducing the medium cost [8,15], PDB was diluted to 50% (dPDB). Glucose supplementation (0–4%) was applied to assess whether sufficient carbon supply could sustain GA production under nitrogen-limited conditions. Compared with PDB without glucose supplementation (Figure 3), dPDB with glucose increased both biomass accumulation and GA content per unit biomass, resulting in higher overall GA production. The highest biomass was observed in dPDB with 4% glucose, while GA production was highest in dPDB with 2% glucose (1167.1 ± 45.2 mg L^−1^ on day 35; Figure 4). These results suggest that combining nitrogen limitation with moderate glucose supplementation supports both mycelial growth and secondary metabolism. Such a metabolic response has been reported to involve increased glycolytic flux and reduced tricarboxylic acid cycle activity under nitrogen-limited conditions, which redirects acetyl-CoA toward the MVA pathway to enhance triterpenoid biosynthesis [15]. Overall, dPDB supplemented with 2% glucose provides a cost-effective medium that maintains high GA production in mycelial cultures.

Despite the typically low expression of key MVA-pathway enzymes in mycelia [1,32], TM701 accumulated exceptionally high GA levels. The four quantified GAs alone surpassed GA contents previously reported for both mycelial and fruiting-body cultures [9,13,14,30,33]. However, because the GA values were expressed as GA-A equivalents, the actual concentrations of individual GA compounds may vary due to differences in molar extinction coefficients. Together with GNPS and volcano plot analyses, these results emphasize the critical influence of strain-specific metabolic regulation on triterpenoid biosynthesis and suggest that intrinsic genetic factors may contribute to the enhanced triterpenoid-producing capacity of TM701.

### 3.4. Effect of Heat Treatment on GA Profile Transformation

**Figure 5:**
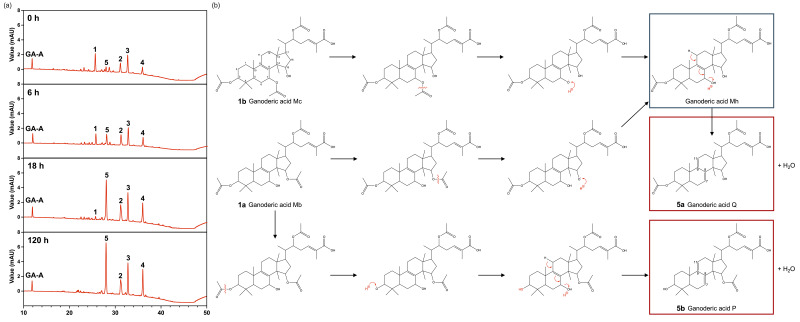
Proposed heat-induced transformation of GAs during heat treatment at 80 °C. (**a**) LC chromatograms of GAs from *G. lucidum* TM701 mycelia after heat treatment at 80 °C for 0 h (control), 6 h, 18 h, and 120 h. Peaks 1–5 were assigned as GA-Mb/GA-Mc, GA-S/GA-Mf, GA-T, GA-R, and GA-P/GA-Q, respectively, based on UPLC-ESI-MS/MS analysis. (**b**) Proposed reaction pathway for the formation of GA-P/GA-Q (peak 5) from GA-Mb/GA-Mc (peak 1). The proposed pathway involves acetyl loss, protonation, and electronic rearrangement, with GA-Mh proposed as a key intermediate.

In industrial processing, harvested mycelia are typically subjected to hot-air drying at 40–80 °C; however, information on the thermal stability of fungal triterpenoids remains limited. To assess potential structural transformations, freeze-dried TM701 mycelia obtained from cultivation in dPDB supplemented with 2% glucose were exposed to 80 °C for up to 120 h. After 18 h of heating, LC chromatograms revealed a marked increase in peak 5 (RT = 27.0 min), identified as GA-P/Q (Figure S5g), accompanied by a decline in GA-Mb/Mc (peak 1), which nearly disappeared (Figure 5a). Other major peaks (peaks 2–4) remained relatively stable. During thermal treatment, MS data revealed that GA-Mh exhibited the most pronounced transient change, with its signal intensity increasing during the initial stage of heating and subsequently decreasing upon prolonged heating (Figure S7). This transient pattern suggests that GA-Mh may serve as an intermediate in the proposed conversion from GA-Mb/Mc to GA-P/Q.

A plausible transformation sequence among GA-Mb/Mc, GA-Mh, and GA-P/Q is proposed in Figure 5b. GAs lacking conjugated dienes (e.g., GA-Mb/Mc) exhibit low absorbance at 245 nm, whereas conjugated dienes in GA-P/Q confer higher UV intensity. Consequently, dehydration and double-bond rearrangement during heating may lead to an apparent increase in GA-P/Q. Similar thermally induced conversions have been reported in plant triterpene saponins, such as ginsenoside dehydration and acetylation reactions during steaming or baking [34,35]. Li et al. [36] demonstrated that GA species bearing double bonds at C-7 and C-9(11) are relatively stable, whereas those with C-8 unsaturation and oxygenation at C-7 readily dehydrate to form conjugated structures. Our observations are consistent with this mechanism, as GA-Mb/Mc species containing C-8 unsaturation may dehydrate to yield the more thermally stable GA-P/Q forms. These findings provide new insights into the chemical stability and possible transformation pathways of lanostane-type triterpenoids under thermal processing.

## 4. Conclusions

Taken together, our results support *G. lucidum* TM701 as a promising mycelial platform for lanostane-type triterpenoid production, reveal extensive metabolite diversity through GNPS-guided metabolomics, and define practical cultivation conditions that balance biomass formation with GA biosynthesis using modest glucose supplementation under nitrogen-limited conditions. The observed heat-induced profile changes suggest a possible interconversion among GA-Mb/Mc → GA-Mh → GA-P/Q, highlighting that the thermal stability of conjugated-diene-bearing GAs is an important consideration for downstream drying, extraction, and quality-control method selection. Although compound quantification was expressed as GA-A equivalents and authentic standards for some triterpenoids remain unavailable, the integrated workflow combining untargeted LC-MS/MS analysis, GNPS-based molecular networking, and targeted GA species quantification provides a useful template for strain screening and process optimization. Future work combining genomics, transcriptomics, and bioreactor scale-up should further elucidate regulatory control points in GA biosynthesis and accelerate the development of standardized GA-rich mycelial ingredients.

## Data Availability

The original contributions presented in this study are included in the article/Supplementary Material. Further inquiries can be directed to the corresponding author.

## Supplementary Materials

The following supporting information can be downloaded at: https://www.mdpi.com/article/10.3390/jof12070500/s1, Figure S1: Appearance of *Ganoderma lucidum* TM701 fruiting bodies cultivated for different durations; Figure S2: Appearance of the second-stage static cultivation of *Ganoderma lucidum* TM701 mycelia; Figure S3: The calibration curve of GA-A; Figure S4: MS/MS molecular network of *Ganoderma lucidum* strains TM701 and BCRC 36203 generated using GNPS; Figure S5: MS/MS identification spectra of triterpenoid species in *Ganoderma lucidum* TM701 mycelia; Figure S6: Changes in biomass, residual sugar, pH, and dissolved oxygen during the first-stage submerged fermentation; Figure S7: Extracted ion chromatograms (EICs) for ganoderic acid Mh (*m*/*z* 587.3605, [M-H]^−^) from LC-ESI-MS/MS analysis of TM701 mycelia at different time intervals during heat treatment at 80 °C; Table S1: BLASTn results of strain TM701 using primers ITS4 and ITS5; Table S2: MS instrument settings and acquisition parameters; Table S3: GAs content of *Ganoderma lucidum* TM701 and BCRC 36203; Table S4: Node IDs and corresponding precursor *m*/*z* values of the main GA cluster identified by GNPS molecular networking analysis.

## Abbreviations

The following abbreviations are used in this manuscript:

ACN	Acetonitrile
DNS	3,5-dinitrosalicylic acid
dPDB	Potato dextrose broth diluted by half
FC	fold change
GAs	ganoderic acids
GNPS	Global Natural Products Social
HMGR	3-hydroxy-3-methylglutaryl coenzyme A reductase
LC	liquid chromatography
ITS	internal transcribed spacer
LS	lanosterol synthase
MVA	mevalonate
PDB	potato dextrose broth
RT	retention time
SD	standard deviation
SQS	squalene synthase
UPLC-ESI-UV-MS/MS	ultra-performance liquid chromatography coupled with electrospray ionization, ultraviolet detection, and tandem mass spectrometry
