# Supplementary material for: Enhanced Production and Profiling of Ganoderic Acids in Ganoderma lucidum Mycelia via Two-Stage Cultivation and GNPS-Guided Metabolomics
Source: J Fungi (Basel). 2026 Jul 8;12(7):500. doi: 10.3390/jof12070500 (PMC13412684; doi:10.3390/jof12070500)
Supplement: Supplementary file 1 [file jof-12-00500-s001.zip › jof-4392699-supplementary.pdf]

Supplementary Information

Enhanced Production and Profiling of Ganoderic Acids in *Ganoderma lucidum* Mycelia via Two-Stage Cultivation and GNPS-Guided Metabolomics

Chieh-Hsi Tsao[1†], Hsin-Ya Tsai[1†], Kai-Wen Cheng[2], Guan-Yuan Chen[3], Hao-Ting Chen[1], Cheng-Chih Hsu[2,4], Nan-Wei Su[1,5*]

[1]Department of Agricultural Chemistry, National Taiwan University, Taipei 106, Taiwan

[2]Leeuwenhoek Laboratories Co. Ltd, 106070, Taipei, Taiwan

[3]Department and Graduate Institute of Forensic Medicine, College of Medicine, National Taiwan University, Taipei 106, Taiwan

[4]Department of Chemistry, National Taiwan University, Taipei 10617, Taiwan

[5]Department of Biochemical Science and Technology, National Taiwan University, Taipei 106, Taiwan

[†]These authors contributed equally to this work

***Corresponding author**

*Nan-Wei Su, Ph.D.

Tel: +886-2-33664806

E-mail: snw@ntu.edu.tw

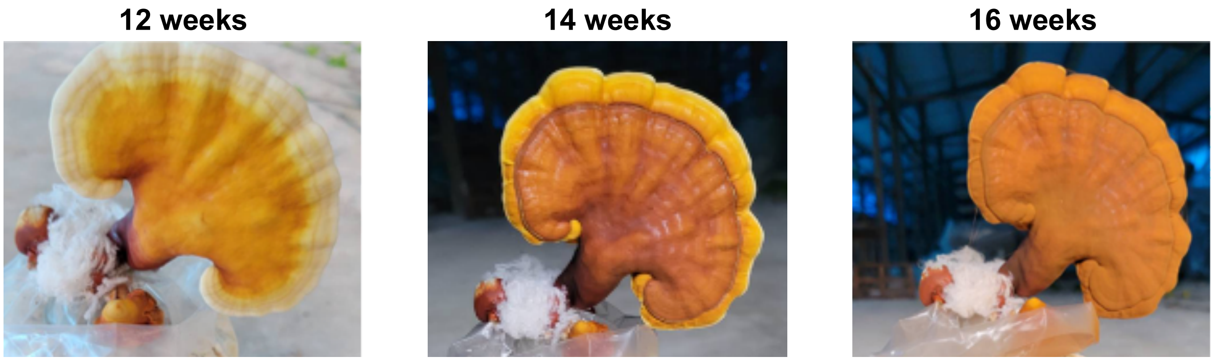

Figure S1. Appearance of *Ganoderma lucidum* TM701 fruiting bodies cultivated for different durations.

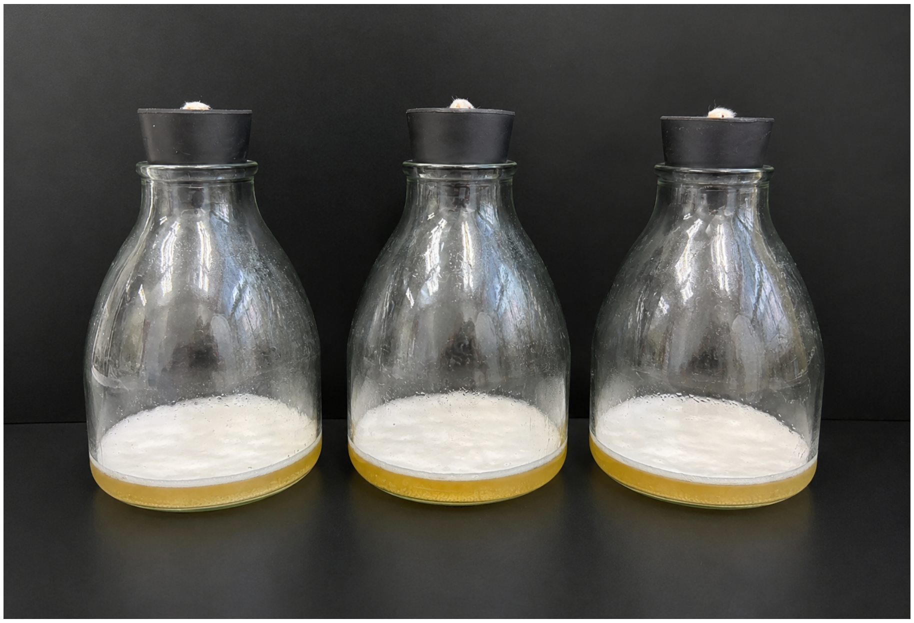

Figure S2. Appearance of the second-stage static cultivation of *Ganoderma lucidum* TM701 mycelia. Cultures were incubated statically in 10-cm-diameter glass jars sealed with cotton plugs to allow gas exchange. The bottles were maintained at 30 °C and 80–85% relative humidity in the dark for 35 days. All experiments were performed with five independent biological replicates (n = 5).

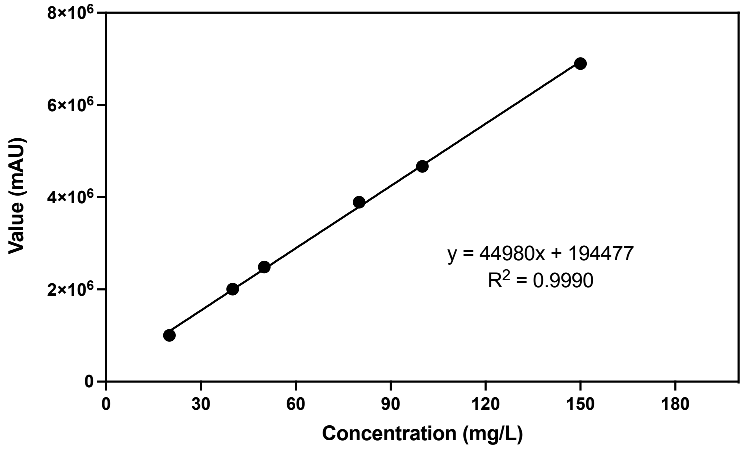

Figure S3. The calibration curve of GA-A.

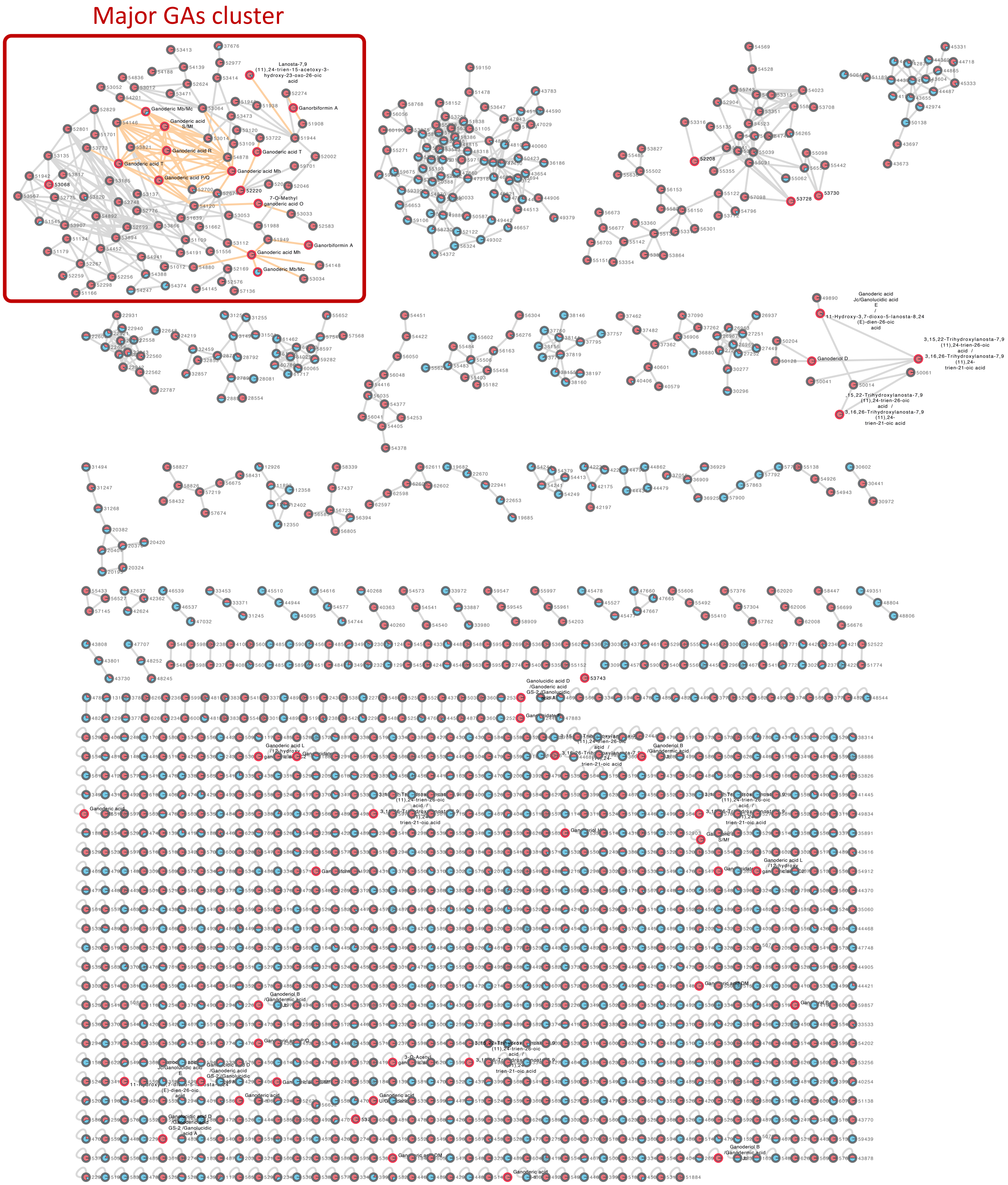

Figure S4. MS/MS molecular network of *Ganoderma lucidum* strains TM701 and BCRC 36203 generated using GNPS. Node color indicates the strains in which the compound was found (coral:

TM701; blue: BCRC 36203). Each node is displayed as a pie chart, with the color proportions reflecting the relative spectral counts contributed by each strain. Red-framed nodes represent identified compounds, with orange edges indicating their spectral similarity. Four nodes (GA-Mb/GA-Mc, GA-S/GA-Mf, GA-T, and GA-R) were selected as representative triterpenoid markers for subsequent experiments. Nodes with the highest structural similarity are positioned closer to the center, reflecting their central role in the cluster's connectivity.

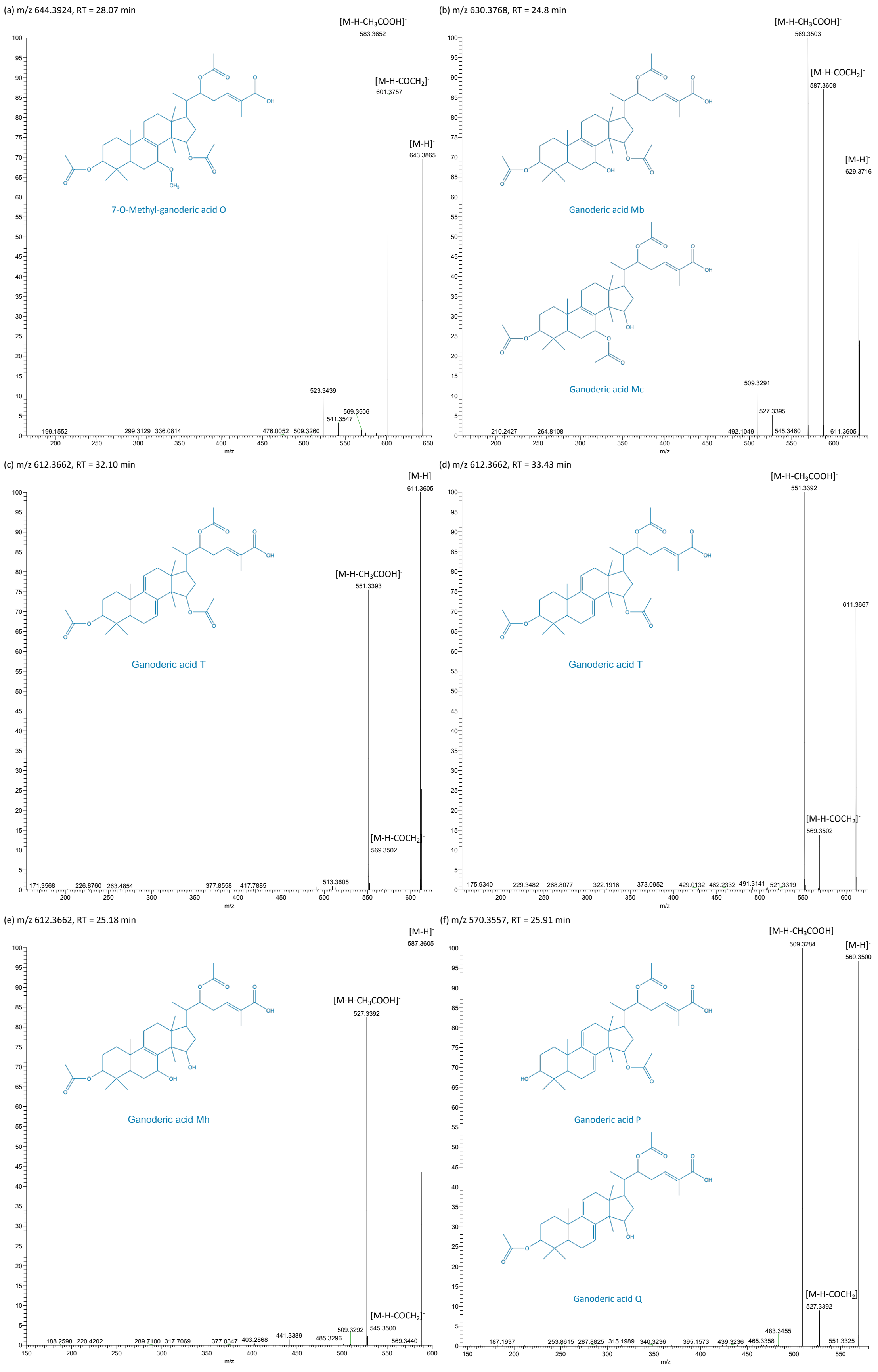

(a) m/z 644.3924, RT = 28.07 min
[M-H-CH3COOH]-
583.3652
[M-H-COCH2]-
601.3757
[M-H]-
643.3865
7-O-Methyl-ganoderic acid O
523.3439
541.3547
569.3506
199.1552
299.3129
336.0814
476.0052
509.3260
(b) m/z 630.3768, RT = 24.8 min
[M-H-CH3COOH]-
569.3503
[M-H-COCH2]-
587.3608
[M-H]-
629.3716
Ganoderic acid Mb
Ganoderic acid Mc
509.3291
527.3395
545.3460
210.2427
264.8108
492.1049
611.3605
(c) m/z 612.3662, RT = 32.10 min
[M-H]-
611.3605
[M-H-CH3COOH]-
551.3393
[M-H-COCH2]-
569.3502
Ganoderic acid T
171.3568
226.8760
263.4854
377.8558
417.7885
513.3605
(d) m/z 612.3662, RT = 33.43 min
[M-H-CH3COOH]-
551.3392
611.3667
[M-H-COCH2]-
569.3502
Ganoderic acid T
175.9340
229.3482
268.8077
322.1916
373.0952
429.0132
462.2332
491.3141
521.3319
(e) m/z 612.3662, RT = 25.18 min
[M-H]-
587.3605
[M-H-CH3COOH]-
527.3392
[M-H-COCH2]-
545.3500
Ganoderic acid Mh
188.2598
220.4202
289.7100
317.7069
377.0347
403.2868
441.3389
485.3296
509.3292
569.3440
(f) m/z 570.3557, RT = 25.91 min
[M-H-CH3COOH]-
509.3284
[M-H]-
569.3500
Ganoderic acid P
Ganoderic acid Q
[M-H-COCH2]-
527.3392
483.3455
187.1937
253.8615
287.8825
315.1989
340.3236
395.1573
439.3236
465.3358
551.3325
m/z

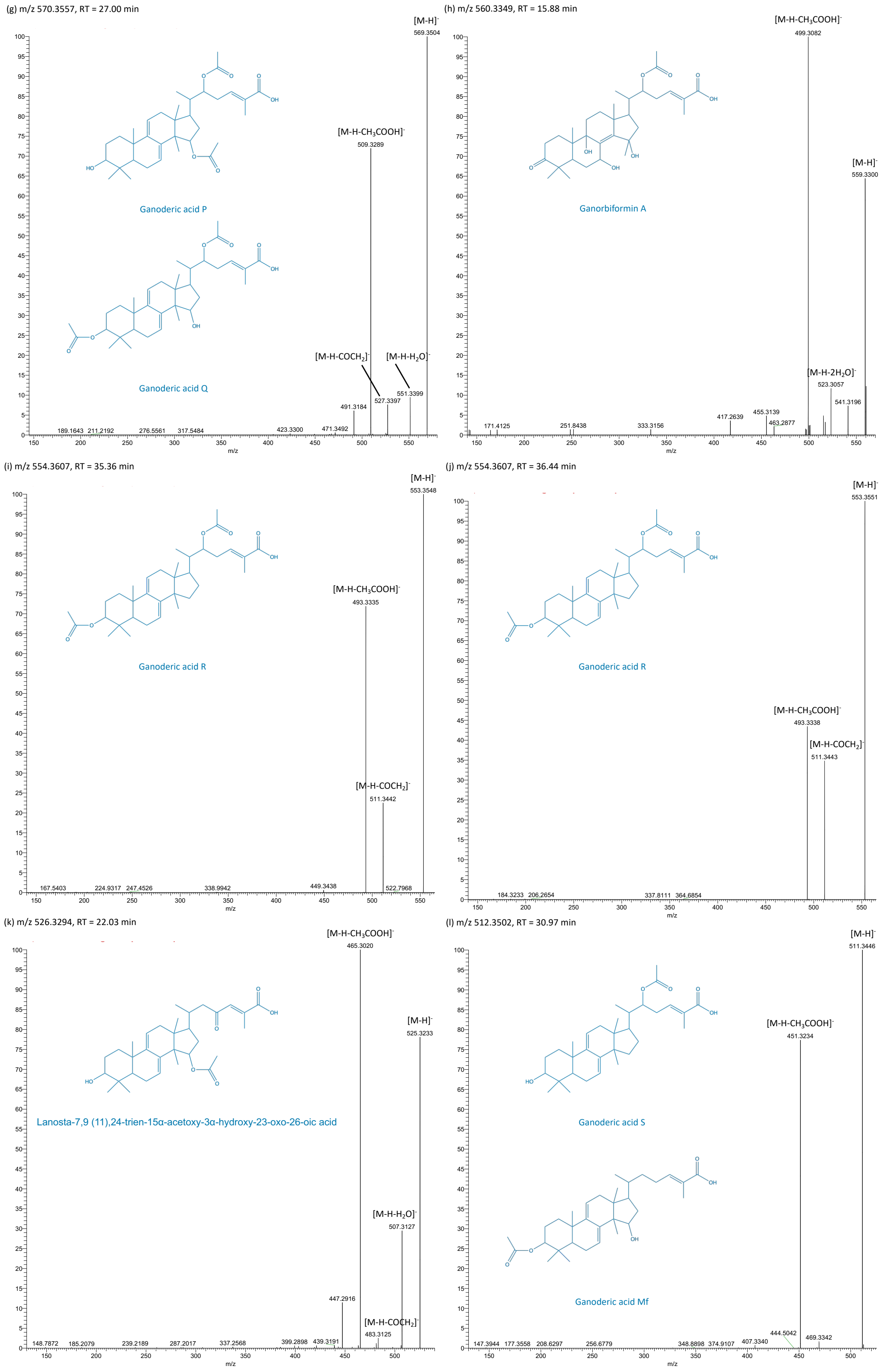

(g) m/z 570.3557, RT = 27.00 min
[M-H]-
569.3504
[M-H-CH3COOH]-
509.3289
[M-H-COCH2]-
527.3397
[M-H-H2O]-
551.3399
491.3184
189.1643 211.2192 276.5561 317.5484 423.3300 471.3492
Ganoderic acid P
Ganoderic acid Q
m/z
(h) m/z 560.3349, RT = 15.88 min
[M-H-CH3COOH]-
499.3082
[M-H]-
559.3300
[M-H-2H2O]-
523.3057
541.3196
171.4125 251.8438 333.3156 417.2639 455.3139 463.2877
Ganorbiformin A
m/z
(i) m/z 554.3607, RT = 35.36 min
[M-H]-
553.3548
[M-H-CH3COOH]-
493.3335
[M-H-COCH2]-
511.3442
167.5403 224.9317 247.4526 338.9942 449.3438 522.7968
Ganoderic acid R
m/z
(j) m/z 554.3607, RT = 36.44 min
[M-H]-
553.3551
[M-H-CH3COOH]-
493.3338
[M-H-COCH2]-
511.3443
184.3233 206.2654 337.8111 364.6854
Ganoderic acid R
m/z
(k) m/z 526.3294, RT = 22.03 min
[M-H-CH3COOH]-
465.3020
[M-H]-
525.3233
[M-H-H2O]-
507.3127
447.2916
[M-H-COCH2]-
483.3125
148.7872 185.2079 239.2189 287.2017 337.2568 399.2898 439.3191
Lanosta-7,9 (11),24-trien-15α-acetoxy-3α-hydroxy-23-oxo-26-oic acid
m/z
(l) m/z 512.3502, RT = 30.97 min
[M-H]-
511.3446
[M-H-CH3COOH]-
451.3234
Ganoderic acid S
Ganoderic acid Mf
147.3944 177.3558 208.6297 256.6779 348.8898 374.9107 407.3340 444.5042 469.3342
m/z

(m) m/z 502.3294, RT = 13.40 min

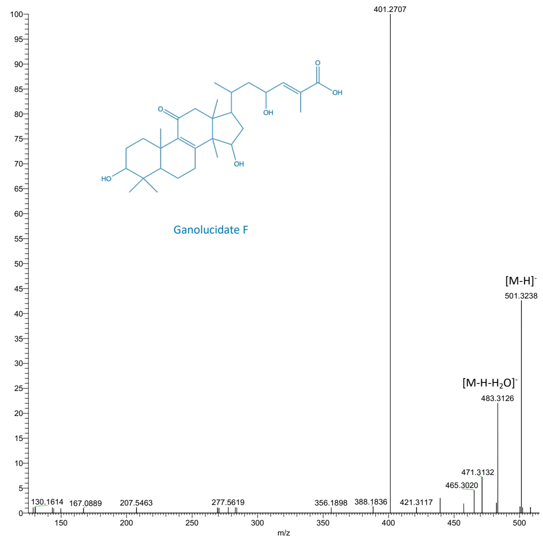

(n) m/z 500.3138, RT = 15.03 min

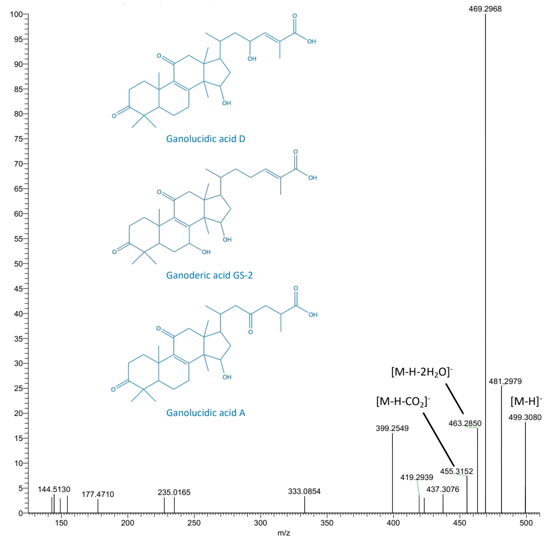

(o) m/z 484.3189, RT = 18.55 min

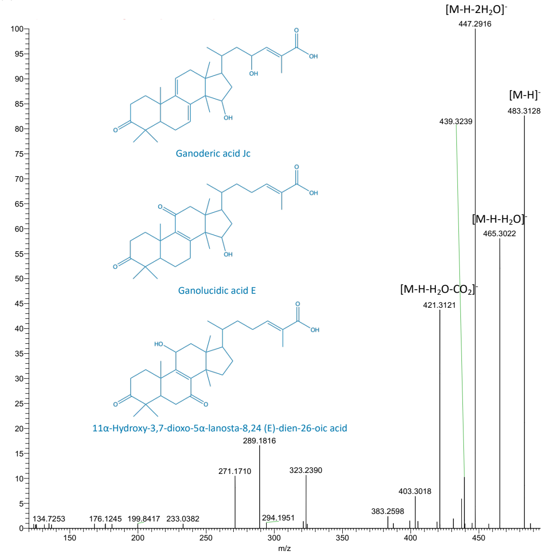

(p) m/z 484.3189, RT = 18.61 min

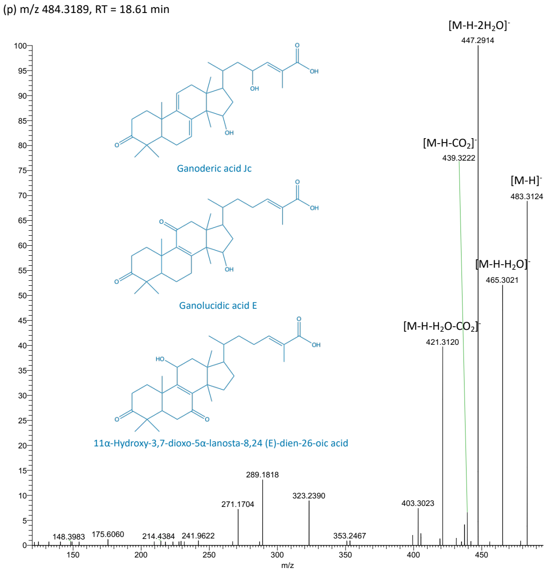

(q) m/z 484.3189, RT = 21.72 min

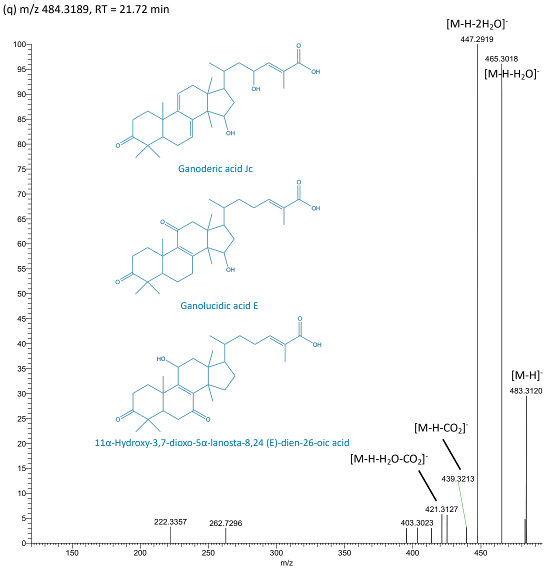

(r) m/z 470.3396, RT = 22.58 min

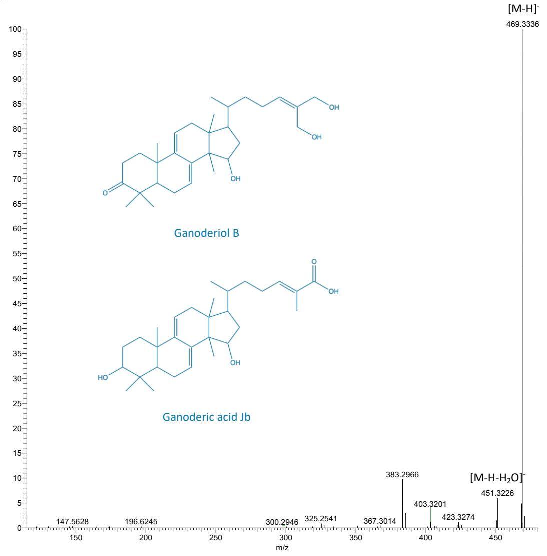

41

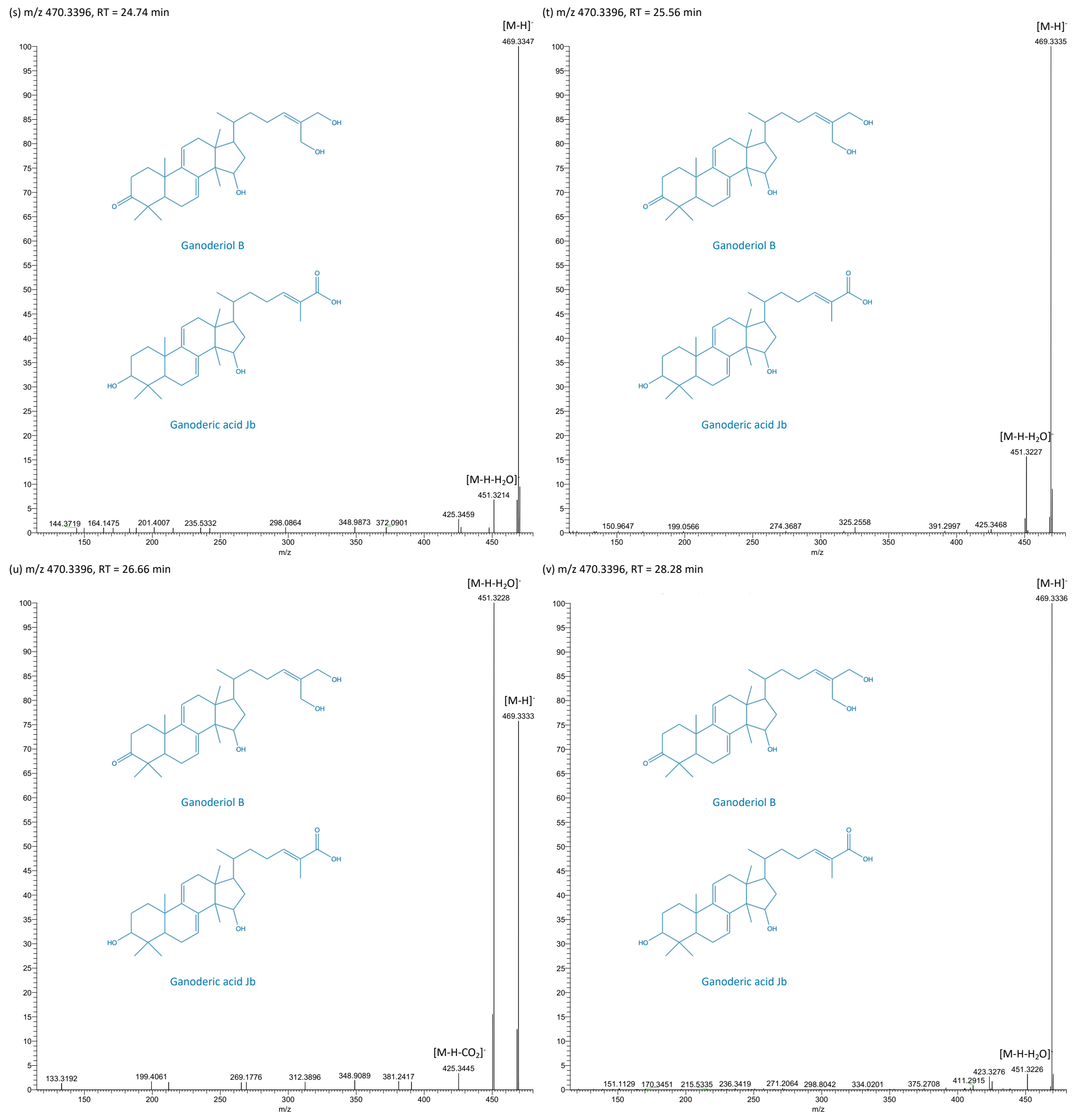

Figure S5. MS/MS identification spectra of triterpenoid species in *Ganoderma lucidum* TM701 mycelia.

(a) 7-O-methyl-ganoderic acid O (RT = 28.07 min),

(b) ganoderic acid Mb/ganoderic acid Mc (RT = 24.80 min),

(c) ganoderic acid T (RT = 32.10 min),

(d) ganoderic acid T (RT = 33.43 min),

(e) ganoderic acid Mh (RT = 25.18 min),

(f) ganoderic acid P/ganoderic acid Q (RT = 25.91 min),

(g) ganoderic acid P/ganoderic acid Q (RT = 27.00 min),

(h) ganorbiformin A (RT = 15.88 min)

(i) ganoderic acid R (RT = 35.36 min),

(j) ganoderic acid R (RT = 36.44 min),

(k) lanosta-7,9 (11),24-trien-15α-acetoxy-3α-hydroxy-23-oxo-26-oic acid (RT = 22.03 min),

(l) ganoderic acid S/ganoderic acid Mf (RT = 30.97 min),

(m) ganolucidate F (RT = 13.40 min),

(n) ganolucidic acid D/ganoderic acid GS-2/ganolucidic acid A (RT = 15.03 min),

(o) ganoderic acid Jc/ganolucidic acid E/11-Hydroxy-GA-Je (RT = 18.55 min),

(p) ganoderic acid Jc/ganolucidic acid E/11-Hydroxy-GA-Je (RT = 18.61 min),

(q) ganoderic acid Jc/ganolucidic acid E/11-Hydroxy-HA-Je (RT = 21.72 min),

(r) ganoderiol B/ganoderic acid Jb (RT = 22.58 min),

(s) ganoderiol B/ganoderic acid Jb (RT = 24.74 min),

(t) ganoderiol B/ganoderic acid Jb (RT = 25.56 min),

(u) ganoderiol B/ganoderic acid Jb (RT = 26.66 min),

(v) ganoderiol B/ganoderic acid Jb (RT = 28.28 min).

11-Hydroxy-HA-Je: 11α-hydroxy-3,7-dioxo-5α-lanosta-8,24-dien-26-oic acid.

Notably, panels (b), (c), (i), and (l) correspond to peaks 1, 3, 4, and 2 in Fig. 1a of the main text, respectively.

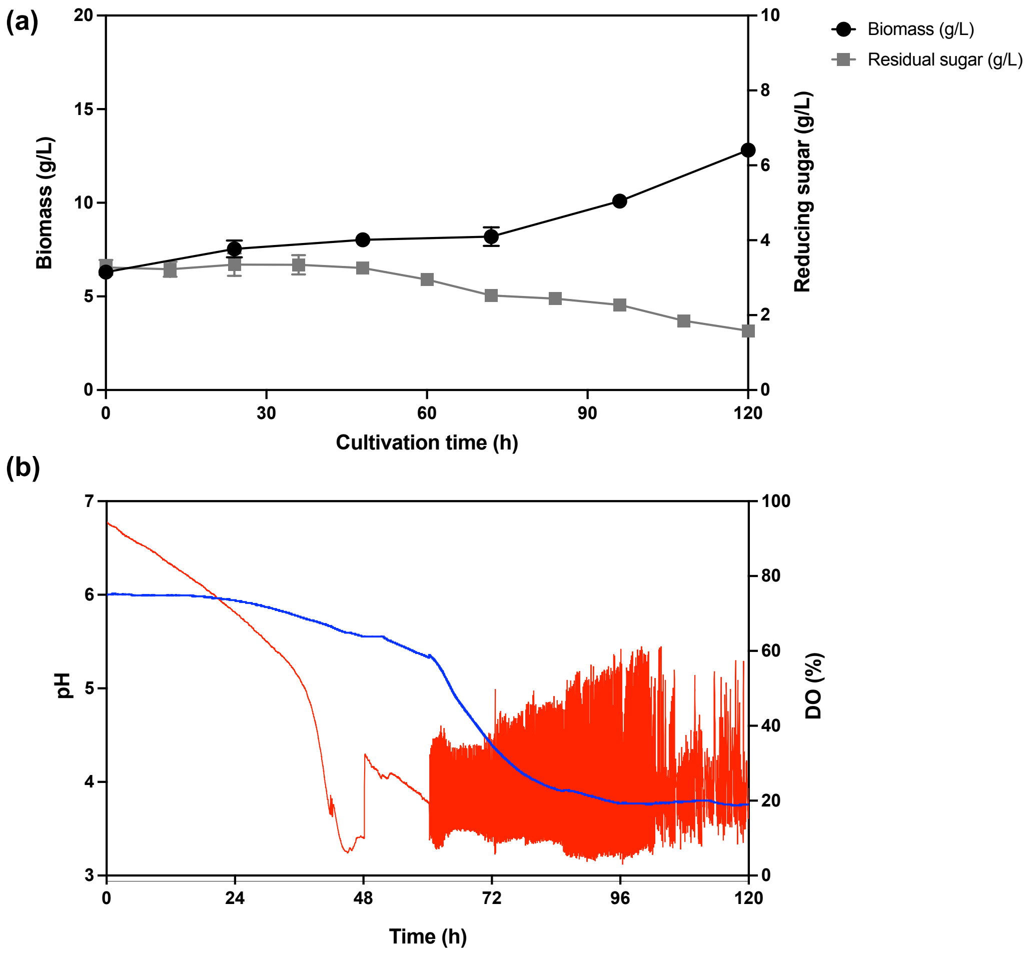

Figure S6. Changes in biomass, residual sugar, pH, and dissolved oxygen (DO) during the first-stage submerged fermentation in the bioreactor. (a) Biomass and residual sugar. (b) pH and dissolved oxygen (DO).

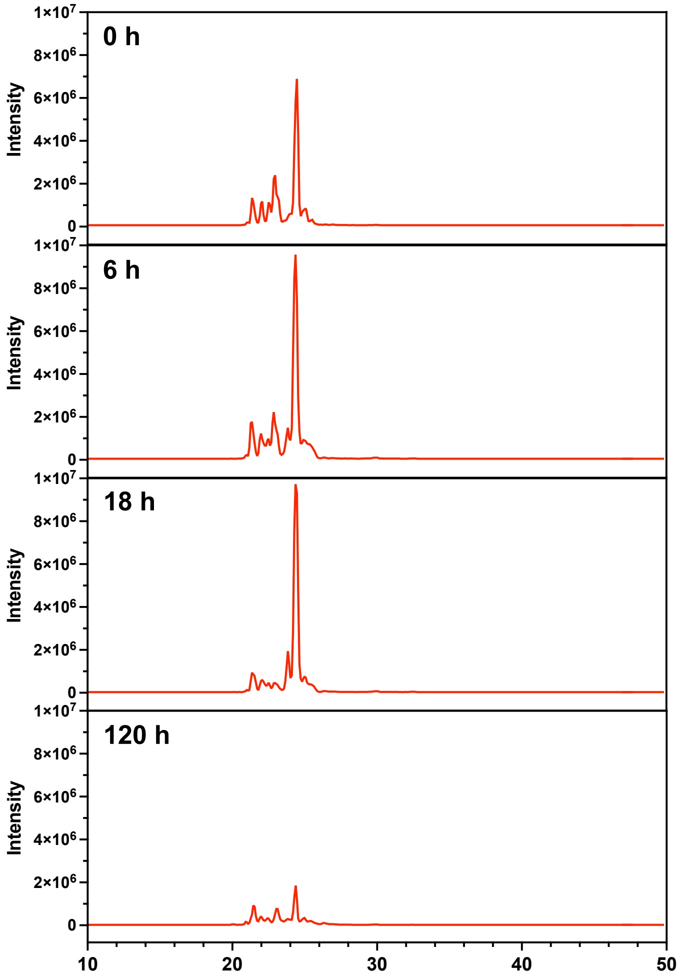

Figure S7. Extracted ion chromatograms (EICs) for ganoderic acid Mh (*m/z* 587.3605, $[M-H]^-$) from LC-ESI-MS/MS analysis of TM701 mycelia at different time intervals during heat treatment at 80°C.

Table S1. BLASTn results of strain TM701 using primers ITS4 and ITS5.

Query sequence	Hit	Id	Description	E-value	Score	Bit score	HSP start	HSP end	HSP length	Query start	Query end	Overlap	%Identity	%Positive	%Gaps
TM701	MF476198	1.34E+09	Ganoderma lucidum isolate 61 small subunit ribosomal RNA gene, partial sequence; internal transcribed spacer 1, 5.8S ribosomal RNA gene, and internal transcribed spacer 2, complete sequence; and large subunit ribosomal RNA gene, partial sequence	0	1254	1132	59	685	627	1	627	100	100	100	0
TM701	JQ781862	4.07E+08	Ganoderma lingzhi voucher Cui6982 18S ribosomal RNA gene, partial sequence; internal transcribed spacer 1, 5.8S ribosomal RNA gene, and internal transcribed spacer 2, complete sequence; and 28S ribosomal RNA gene, partial sequence	0	1254	1132	18	644	627	1	627	100	100	100	0
TM701	JQ781861	4.07E+08	Ganoderma lingzhi voucher Dai12438 18S ribosomal RNA gene, partial sequence; internal transcribed spacer 1, 5.8S ribosomal RNA gene, and internal transcribed spacer 2, complete sequence; and 28S ribosomal RNA gene, partial sequence	0	1254	1132	6	632	627	1	627	100	100	100	0
TM701	JQ781860	4.07E+08	Ganoderma lingzhi voucher Dai10631 18S ribosomal RNA gene, partial sequence; internal transcribed spacer 1, 5.8S ribosomal RNA gene, and internal transcribed spacer 2, complete sequence; and 28S ribosomal RNA gene, partial sequence	0	1251	1129.29	7	633	627	1	627	100	99.84051	99.84051	0
TM701	MF476200	1.34E+09	Ganoderma lucidum isolate 49 small subunit ribosomal RNA gene, partial sequence; internal transcribed spacer 1, 5.8S ribosomal RNA gene, and internal transcribed spacer 2, complete sequence; and large subunit ribosomal RNA gene, partial sequence	0	1249	1127.49	61	687	627	1	627	100	99.84051	99.84051	0
TM701	MF476197	1.34E+09	Ganoderma lucidum isolate 32 small subunit ribosomal RNA gene, partial sequence; internal transcribed spacer 1, 5.8S ribosomal RNA gene, and internal transcribed spacer 2, complete sequence; and large subunit ribosomal RNA gene, partial sequence	0	1249	1127.49	13	639	627	1	627	100	99.84051	99.84051	0
TM701	KT693255	1E+09	Ganoderma sichuanense isolate B1_1406706 clone 2 18S ribosomal RNA gene, partial sequence; internal transcribed spacer 1, 5.8S ribosomal RNA gene, and internal transcribed spacer 2, complete sequence; and 28S ribosomal RNA gene, partial sequence	0	1249	1127.49	11	637	627	1	627	100	99.84051	99.84051	0
TM701	OR272196	2.55E+09	Ganoderma sichuanense isolate GKR7 small subunit ribosomal RNA gene, partial sequence; internal transcribed spacer 1, 5.8S ribosomal RNA gene, and internal transcribed spacer 2, complete sequence; and large subunit ribosomal RNA gene, partial sequence	0	1249	1127.49	6	632	627	1	627	100	99.84051	99.84051	0

Query sequence	Hit	Id	Description	E-value	Score	Bit score	HSP start	HSP end	HSP length	Query start	Query end	Overlap	%Identity	%Positive	%Gaps
TM701	ON256156	2.23E+09	Ganoderma lucidum isolate Univ.M1 small subunit ribosomal RNA gene, partial sequence; internal transcribed spacer 1, 5.8S ribosomal RNA gene, and internal transcribed spacer 2, complete sequence; and large subunit ribosomal RNA gene, partial sequence	0	1249	1127.49	9	635	627	1	627	100	99.84051	99.84051	0
TM701	JQ781865	4.07E+08	Ganoderma lingzhi voucher IFP01021 18S ribosomal RNA gene, partial sequence; internal transcribed spacer 1, 5.8S ribosomal RNA gene, and internal transcribed spacer 2, complete sequence; and 28S ribosomal RNA gene, partial sequence	0	1249	1127.49	5	631	627	1	627	100	99.84051	99.84051	0
TM701	JQ781855	4.07E+08	Ganoderma lingzhi voucher Dai 12573 18S ribosomal RNA gene, partial sequence; internal transcribed spacer 1, 5.8S ribosomal RNA gene, and internal transcribed spacer 2, complete sequence; and 28S ribosomal RNA gene, partial sequence	0	1249	1127.49	42	668	627	1	627	100	99.84051	99.84051	0
TM701	FJ940919	2.82E+08	Ganoderma lucidum strain CSAAS0801 18S ribosomal RNA gene, partial sequence; internal transcribed spacer 1, 5.8S ribosomal RNA gene, and internal transcribed spacer 2, complete sequence; and 28S ribosomal RNA gene, partial sequence	0	1249	1127.49	9	635	627	1	627	100	99.84051	99.84051	0
TM701	JQ781856	4.07E+08	Ganoderma lingzhi voucher Cui4018 18S ribosomal RNA gene, partial sequence; internal transcribed spacer 1, 5.8S ribosomal RNA gene, and internal transcribed spacer 2, complete sequence; and 28S ribosomal RNA gene, partial sequence	0	1247	1125.69	3	628	626	1	626	99.84051	99.84026	99.84026	0
TM701	MN431189	1.83E+09	Ganoderma lucidum small subunit ribosomal RNA gene, partial sequence; internal transcribed spacer 1, 5.8S ribosomal RNA gene, and internal transcribed spacer 2, complete sequence; and large subunit ribosomal RNA gene, partial sequence	0	1246	1124.79	6	628	623	5	627	99.36204	100	100	0

83

Table S2. MS instrument settings and acquisition parameters.

**Source type**	**HESI**
Capillary Temp (°C)	320
Source Heater Temp (°C)	280
Sheath Gas Flow	60
Aux Gas Flow	10
Source Voltage (kV)	4
Polarity	Negative
Scan type	Full scan
Resolution	60000
Automatic gain control	1E+06
Maximum injection time (ms)	100
Scan range (m/z)	70-1000
	Top10 (ddMS2)
Resolution	15000
AGC	5E+04
Maximum injection time (ms)	150
Isolation window [m/z]	2
Normalized Collision Energy	25
Dynamic exclusion	60
Minimum AGC	500

Table S3. GAs content of *Ganoderma lucidum* TM701 and BCRC 36203. Data are presented as mean ± SD (n = 5).

	**TM701**	**BCRC 36203**
GA content (mg g^{-1} DW)	87.93	12.60
GA production (mg L^{-1})	360.23	31.42

The culture broth containing mycelia from the first-stage cultivation was used as the inoculum and transferred at 10% (v/v) into sterilized jars containing 45 mL of PDB, followed by incubation at 30 °C and 80–85% relative humidity for 35 days.

Table S4. Node IDs and corresponding precursor m/z values of the main GA cluster identified by GNPS molecular networking analysis.

Node IDs	Precursor m/z
37676	273.099
51012	510.332
51048 (Ganoderic acid S/Mf)	511.344
51109	513.358
51134	514.359
51166	515.366
51179	516.369
51466 (Lanosta-7,9 (11),24-trien-15α-acetoxy-3α-hydroxy-23-oxo-26-oic acid)	525.323
51545	528.342
51556	529.354
51639	530.358
51662	531.363
51701	532.363
51908	541.318
51938	543.255
51940	543.287
51942	543.328
51944	543.334
51949	543.334
51988	544.337
52002	545.348
52023	545.351
52046	546.354
52169	551.338
52217 (Ganoderic acid R)	553.355
52220	553.355
52256	555.364
52259	555.370
52267	556.368
52274	557.313
52298	557.377
52323 (Ganorbiformin A)	559.329
52328 (Ganorbiformin A)	559.329
52576	567.332

Table S4. *(continued)*

Node IDs	Precursor m/z
52583	567.370
52624	569.303
52633 (Ganoderic acid P/Q)	569.349
52674	570.353
52699	571.355
52700	571.365
52748	572.369
52775	573.371
52776	573.372
52801	574.374
52829	575.377
52977	583.329
53012	585.298
53014	585.344
53033	585.381
53034	585.381
53052	586.301
53053	586.348
53064	587.314
53068	587.357
53069 (Ganoderic acid Mh)	587.360
53077 (Ganoderic acid Mh)	587.360
53109	588.317
53112	588.363
53120	589.310
53135	589.367
53137	589.368
53185	590.369
53413	601.293
53414	601.340
53471	603.309
53473	603.355
53567	605.362
53722	611.316
53738 (Ganoderic acid T)	611.360
53740 (Ganoderic acid T)	611.360
53773	612.364
53817	613.340

Table S4. *(continued)*

Node IDs	Precursor m/z
53820	613.367
53821	613.376
53866	614.369
53894	615.382
53907	616.385
54120	625.376
54139	627.308
54145	627.354
54146	627.355
54148	627.353
54188	628.312
54191	628.358
54201	629.306
54214 (Ganoderic Mb/Mc)	629.371
54215 (Ganoderic Mb/Mc)	629.371
54247	630.863
54374	631.173
54388	631.377
54452	633.383
54836	643.304
54852 (7-O-Methyl ganoderic acid O)	643.386
54878	645.366
54880	645.366
54892	645.393
54941	647.372
57136	727.321
59701	826.562
